# B-cell activating factor BAFF as a novel alert marker for the immunological risk stratification after kidney transplantation

**DOI:** 10.1007/s12026-021-09205-4

**Published:** 2021-08-10

**Authors:** Antonia Margarete Schuster, N. Miesgang, L. Steines, C. Bach, B. Banas, T. Bergler

**Affiliations:** 1 Department of Nephrology, University Hospital Regensburg, Franz-Josef-Strauß-Allee 11, 93053 Regensburg, Germany; 2 Department of Internal Medicine 5, University Hospital Erlangen, Erlangen, Germany

**Keywords:** BAFF, ABMR, Immunological risk stratification, Kidney transplantation

## Abstract

The B cell activating factor BAFF has gained importance in the context of kidney transplantation due to its role in B cell survival. Studies have shown that BAFF correlates with an increased incidence of antibody-mediated rejection and the development of donor-specific antibodies. In this study, we analyzed a defined cohort of kidney transplant recipients who were treated with standardized immunosuppressive regimens according to their immunological risk profile. The aim was to add BAFF as an awareness marker in the course after transplantation to consider patient’s individual immunological risk profile. Included patients were transplanted between 2016 and 2018. Baseline data, graft function, the occurrence of rejection episodes, signs of microvascular infiltration, and DSA kinetics were recorded over 3 years. BAFF levels were determined 14 d, 3 and 12 months post transplantation. Although no difference in graft function could be observed, medium-risk patients showed a clear dynamic in their BAFF levels with low levels shortly after transplantation and an increase in values of 123% over the course of 1 year. Patients with high BAFF values were more susceptible to rejection, especially antibody-mediated rejection and displayed intensified microvascular inflammation; the combination of high BAFF + DSA puts patients at risk. The changing BAFF kinetics of the medium risk group as well as the increased occurrence of rejections at high BAFF values enables BAFF to be seen as an awareness factor. To compensate the changing immunological risk, a switch from a weaker induction therapy to an intensified maintenance therapy is required.

**Supplementary Information:**

The online version contains supplementary material available at 10.1007/s12026-021-09205-4.

## Introduction

The B cell activating factor BAFF plays a crucial role in the development and survival of B-lymphocytes. Because of this role, BAFF gained attention in recent years, also in the context of kidney transplantation. B-lymphocytes make a decisive contribution to the development of donor-specific antibodies through their regulatory functions such as cytokine production, but especially through their conversion into antibody-producing cells [1]. They are therefore of central importance in the context of rejection, mainly antibody-mediated rejection, which is a major cause of renal allograft dysfunction and subsequent graft loss [2, 3].

BAFF, also known as BLyS (B lymphocyte stimulator), TALL1 (TNF and apoL-related leukocyte- overexpressed ligand 1), or TNFSF13B (TNF superfamily member 13B) belongs to the TNF superfamily [4] and could be detected in both a soluble and a membrane-bound form.

Different cells express BAFF, including neutrophils, monocytes, macrophages, and dendritic cells. IL-10, IFN-γ, and IFN-α are considered a stimulus of BAFF expression, whereas IL-4 tends to inhibit BAFF expression [5]. BAFF attaches to three known receptors, transmembrane activator and CAML interactor (TACI), B cell maturation antigen (BCMA), and BAFF receptor (BAFF-R or BR3), which are expressed at different times in B cell development and proliferation [6].

By stimulating pro-survival oncogenes like Bcl-2, BAFF is crucial for the survival and proliferation of B-lymphocytes. In mouse models it was shown that the development of mature B lymphocytes in particular is dependent on BAFF and that without BAFF stimulation there is a rapid loss of B cells [6, 7].

Also in the context of autoimmune diseases such as lupus erythematosus and Sjogren’s syndrome, it is assumed that BAFF is relevant due to overexpression and thereby formation of autoreactive B cells [7], and since 2011 belimumab, a humanized monoclonal antibody against BAFF was approved [8].

In the context of transplantation, there are still inconsistent data concerning the relevance of BAFF. In their study, Thibault-Espitia et al. were able to show that increased BAFF levels after transplantation are associated with an increased occurrence of de novo donor-specific antibodies and graft dysfunction [9]. However, this could not be reproduced in other studies. In a previous work, we were able to show that BAFF reflects the immunization status (no PRA vs. verifiable PRA) of patients before transplantation and that patients with higher BAFF values in the follow-up showed an increased risk for an impaired graft function [10]. It was also demonstrated that increased BAFF levels go hand in hand with an increased occurrence of rejections, especially antibody-mediated rejections [11]. In an experimental KTx model in rats, our group showed that chronic underdosing of immunosuppression — analogous to the non-adherence of transplanted patients — induced expression of BAFF and BAFF receptor within allografts [12] and that when a monoclonal anti-BAFF antibody was applied in the that rodent KTx model, DSA development was partially inhibited [13].

BAFF has been used as a therapeutic target in a phase 2a study. Usage of the BAFF inhibitor tabalumab in pre-immunized patients with end-stage renal disease resulted in a reduction of the cPRA levels [14]. In a more clinically relevant setting, Banham et al. conducted a double-blind, randomized, placebo-controlled phase 2 study in which kidney transplant patients were treated with an anti-BAFF antibody, belimumab, or placebo after transplantation. The aim of the study was a more detailed analysis of the influence of belimumab on IgG production and on B cell homeostasis. Despite similar rates of adverse events in both groups, the co-primary endpoint of a reduced naïve B-cell amount was not achieved [15].

The aim of our current study was to analyze a pre-defined highly standardized cohort of kidney transplant patients, who were treated with a specific immunosuppressive regimen according to their immunological risk profile, with regard to the development of BAFF levels. The immunological risk profile of kidney transplant recipients is currently defined by clinical parameters such as PRA level, presence of DSA, development of de novo DSA, and re-transplantation and determines the choice of immunosuppressive induction and maintenance therapy. This is also reflected in the current KDIGO guidelines. Here, the patients are stratified into a low, medium, and high-risk group based on immunology, and both induction and maintenance therapy are then adapted accordingly. Apart from such clinical factors, functional T cell assays, such as a specific ELISpot testing, in the context of CMV infections, are available. However, the possibility of a reliable B cell monitoring is currently not established on a routine basis. Given the important functions of B cells [1], this is especially necessary in the transplant setting. Based on previous experience with BAFF, it may be possible to further develop BAFF as an immunological alert marker in the course of kidney transplantation with the aim of administering a tailored immunosuppression for each patient.

Besides monitoring BAFF in the follow-up in correlation to recipients histopathological graft lesions, the occurrence of rejection episodes (TCMR, AMR) and the development of de novo donor-specific antibodies were examined.

## Material and methods

### Patients’ baseline characteristics

Initially, all patients who were transplanted at our center from January 1st, 2016, to December 31th, 2018, were identified (n = 122). Based on their immunological risk profile, the patients were divided into three, pre-defined groups before transplantation: low, medium, or high immunological risk. Both induction therapy and maintenance immunosuppression were then aligned according to this risk stratification. A low immunological risk was defined as an AB0-compatible first transplant, a CDC-PRA level of less than 5% and no detectable anti-HLA- (HLA-A;-B;-C;-DR;-DP;-DQ) or donor-specific antibodies. Patients with a CDC-PRA level of 5–30% and/or detectable anti-HLA antibodies in the absence of donor-specific antibodies or patients with a previous transplant but without immunological graft loss within 2 years were stratified into the medium risk group. Once any donor-specific antibodies could be detected (MFI > 500) or any CDC-PRA level above 30% and patients have suffered from an early immunological graft loss in a previous transplant, they were assigned to the high risk group (Supplement Table 1). The sum of HLA-mismatches was not taken into account when classifying the immunological risk. In our cohort, there were 119 Caucasian (97.5%) and 3 patients of non-Caucasian origin (2.5%). Because of this fact, race was not taken into account in the distribution of the immunological risk. According to the German transplantation law, a donation after cardiac death (DCD) is not permitted, so that all post-mortem donations were made through donations after brain death (DBD). Out of the 122 transplants performed, 41 were from a living donation (33.6%) from which 17 were from blood relatives (41.5%). Forty-nine patients received an organ from a donor with extended donor criteria (ECD) (40.2%).

A total of 122 patients were enrolled, with 44 patients in the low immunological risk group (36.1%), 34 patients in the medium immunological risk group (27.9%), and 44 patients in the high immunological risk group (36.1%). Low- or medium-risk patients received induction therapy with a CD 25 monoclonal antibody basiliximab (Novartis), a therapy that does not deplete T cells but has an immunomodulatory effect. In contrast, high-risk patients were induced with thymoglobulin in a routinely used dose (6 mg/kg body weight) (Sanofi). The maintenance immunosuppressive therapy was in all three groups based on a calcineurin inhibitor (tacrolimus) in combination with a proliferation inhibitor (mycophenolat mofetil or mycophenolat acid) and steroids.

Depending on the pre-defined risk profile, the respective medication was reduced in the follow-up after transplantation. Depending on the underlying renal disease, steroids were stopped after three months or maintained at a dose of 5 mg. The tacrolimus target levels were the same among the three groups. Shortly after transplantation, a target level of 12 was aimed for and was reduced to 8–10 µg/l by the end of month 3. The target level for months 4–12 was 6–8 µg/l and after month 12 a target level of 4–6 µg/l was maintained. The differences in the immunosuppressive regime were determined on the one hand by the induction therapy and on the other hand by the dosages in mycophenolate acid and steroids. The exact details between the immunosuppressive regimens can be found in the supplement in Table 2.

At our center, post-transplant monitoring is carried out according to a predefined standard, as part of the so-called Regensburger Transplantationsnachsorge. Furthermore, all relevant transplant-related data and donor data are archived. Human tissue was analyzed according to the approval of the Ethics Committee of the Medical Faculty of the University of Regensburg.

In addition to the analysis of the baseline data, clinical endpoints such as graft function represented by creatinine, eGFR, and albuminuria over an observation period of 3 years and dosage of the immunosuppression were recorded. The BAFF levels of all patients at 14 days, 3 months, and 12 months were then determined by ELISA.

### BAFF-ELISA analysis

BAFF levels in patients’ sera were measured by using the human Baff/BlyS/TNFSF13 B immunoassay (R&D Systems, Minneapolis, USA) according to the manufacturer’s recommendations. The minimum detectable concentration was 62.5 pg/ml and OD-measurement was done with a Tecan reader (Männedorf, Switzerland).

### Histopathological analysis

Furthermore, we analyzed allograft biopsies (protocol biopsy 14 d and 3 months, as well as indication biopsies during the observation period) with regard to rejection episodes according to the BANFF 2017 criteria’s as well as in respect to allograft lesion score (tubulitis, peritubular capillaritis, etc.) [16]. Additionally, the de novo DSA kinetics (3 and 12 months postTx) were examined. All these parameters were compared first with the underlying immunological risk classification, secondly in regard to the BAFF median. The BAFF median at 14 days was calculated based on all BAFF values of the 122 patients at 14 days.

### Detection of de novo donor-specific antibodies

Antibody tests were carried out according to the standards of the European Federation for Immunognetics (EFI). All patients were routinely tested for donor-specific antibodies prior to transplantation (preTxDSA) and at 3 and 12 months after transplantation (postTxDSA). Specifically, patients’ sera were tested for presence/absence of anti-HLA antibodies using LABScreen® Mixed Assay (LSmixed, One Lambda, Inc, 22,801 Roscoe Blvd. West Hills, CA 91,304, USA), applying the manufacturer's recommended positive test ratio of > 2.2. For positive sera, anti-HLA antibody specificity was determined using a single antigen assay for HLA class I (i.e., HLA-A/B/Cw) and/or HLA class II antigens (i.e., HLA-DR/DQ/DP; LABScreen® Single Antigen Assay, LS1A04 and LS2A01, One Lambda), according to the manufacturer’s instructions. Positive results for antibody specificities in single antigen test were defined by a baseline normalized mean fluorescence intensity (MFI) > 500. All antibody tests were analyzed on a LABScan 200® flow analyzer (One Lambda). Finally, donor-specific HLA-antibodies were determined via comparison of the assigned specificities with the donor HLA-type.

### Statistical analysis

Continuous variables are presented as mean $\pm$ standard deviation, whereas categorical data are shown as frequency distributions (n) and percentages (%). A statistical analysis was performed by the Student’s t-test with a p value < 0.05 indicating a statistical significance. The analysis was calculated using Excel 2016.

## Results

### Stratification in terms of the basic immunological risk

#### Patients’ baseline characteristics

As mentioned above, 122 patients were stratified into the three immunological risk groups (Table 1). No donor nor any recipient derived baseline marker differed between the groups. Regarding immunological aspects, patients in the low-risk group were noticed by significantly more HLA-B mismatches than the other two cohorts (p = 0.03 resp. 0.04). According to the underlying stratification, patients with a high immunological risk showed significantly higher CDC-PRA levels (30%) than the low-risk (0%, p = 1.7 × 10^−6^) and medium-risk group (10%; p = 0.008). Low-risk patients had a significantly shorter cold ischemia time (CIT) than medium-risk patients (p = 0.04). There was no difference in terms of warm ischemia time (WIT). Detailed information concerning the baseline data is shown in Table 1.

**Table 1:** Baseline characteristics and follow- up parameter of renal transplant recipients stratified for immunological risk for allograft rejection

	Low risk (n = 44)	Medium risk (n = 34)	High risk (n = 44)
Donor— age (years)	54 ± 15	55 ± 12	51 ± 19
Donor— weight (kg)	77 ± 18	80 ± 15	78 ± 22
Donor— height (cm)	171 ± 12	174 ± 9	170 ± 17
Donor— sex (M:F)	15:29	21:13	21:23
Recipient — age	55 $\pm$ 12	50 ± 13	54 ± 12
Recipient— weight (kg)	76 ± 10	83 ± 17	77 ± 16
Recipient — height (cm)	172 ± 8	174 ± 9	170 ± 10
Recipient — sex (M:F)	33:11	23:11	29:15
Re-Tx (n)	2	0	6
Duration of RRT; years	3.6 ± 3.7	5.1 ± 4.0	5.3 ± 3.9
Causes of end stage renal disease			
ADPKD	9	6	5
IgA- Nephropathy	6	7	11
Hypertensive	13	5	5
Diabetic	4	2	5
Others	12	14	18
HLA-mismatch			
HLA-A	0.9	0.8	0.7
HLA-B	1.4^a; b^	0.9^a^	1^b^
HLA-DR	1.2	0.9	1
PRA (%) — current	0^b^	2^c^	15^b;c^
PRA (%) — highest	0^a^^;b^	10^a^^;c^	30^b;c^
Ischemia time			
CIT (h/min)	6.5/29^a^	7.8/21^a^	8.5/27
WIT (min)	47	42	45
Rejection episodes (n)			
TCMR	6	0	7
AMR	2	1	2
Borderline	1	2	1
De novo donor specific antibodies			
HLA class I (n/%)	0/0	0/0	6/13.6
HLA class II (n/%)	2/4.5	3/8.8	5/11.4
Graft loss (n/%)	3/6.8	1/2.9	1/2.3
Death (n/%)	3/6.8	0/ 0	2/4.5

^a^Low vs. medium: p < 0.05. ^b^Low vs. high: p < 0.05, ^c^medium vs. high: p < 0.05

The follow-up time in the low-risk group was 20.5 months, in the medium group 22.3 months, and in the high-risk group 18.3 months on average.

#### Immunosuppressive therapy

Concerning the immunosuppression used, it is shown, as prescribed by the stratification, that the patients at high risk were induced significantly more frequently with thymoglobulin, whereas the other patients received basiliximab.

Fourteen days after transplantation, the high-risk patients had higher doses of mycophenolate acid than the two comparison groups. The level of significance was reached each time (low vs. high: p = 0.001 and medium vs. high: p = 0.01). Then, after 3 months, the high-risk population was treated with intensified doses of steroids (p = 0.03 (vs. low risk) and p = 0.009 (vs. medium risk). No further differences in immunosuppressive therapy could be demonstrated. Detailed information concerning the immunosuppressive therapy is shown in Table 2.

**Table 2:** Real life immunosuppressive doses and target levels of the three cohorts during follow-up until month 12 after transplantation

	Low risk (n = 44)			Medium risk (n = 34)			High risk (n = 44)		
	14 days postTX	3 months postTX	12 months post Tx	14 days postTX	3 months postTX	12 months post Tx	14 days postTX	3 months postTX	12 months post Tx
Calcineurin-inhibitor^*^	11.3 $\pm$ 2.9	9.0 $\pm$ 1.9	6.7 $\pm$ 2.2	10.5 $\pm$ 2.7	9.1 $\pm$ 2.8	7.3 $\pm$ 3.9	10.7 $\pm$ 2.5	9.2 $\pm$ 2.3	7.0 $\pm$ 2.1
Mycophenolat acid (mg)	1236.2 $\pm$ 356.5^b^	1046.7 $\pm$ 429.7	743.6 $\pm$ 449.8^a^	1283.5 $\pm$ 332.8^c^	1035.8 $\pm$ 369.0	971.4 $\pm$ 382.5^a^	1450 $\pm$ 224^b;c^	1202.5 $\pm$ 396.8	943.2 $\pm$ 401.1
Steroids (mg)	14.7 $\pm$ 4.1	5.7 $\pm$ 3.2^b^	2.9 $\pm$ 5.5	14.7 $\pm$ 2.7	5.8 $\pm$ 1.6^c^	3.2 $\pm$ 2.3^c^	16.2 $\pm$ 4.1	7.1 $\pm$ 2.2^b:c^	4.5 $\pm$ 1.6^c^

^*^The measured target levels are given here. ^a^Low vs. medium: p < 0.05. ^b^Low vs. high: p < 0.05, ^c^medium vs. high: p < 0.05

#### Resulting allograft function

With regard to the clinical course after transplantation, creatinine with the corresponding eGFR (CKD-EPI) and the albuminuria at the time points 14 d, 3 and 12 months, and after 2 and 3 years were analyzed. There was no statistically significant difference detectable between the three groups at any of these time points (Supplement Table 3). In further analyzes, we could see that even in the stratification according to BAFF median, no influence on graft function (creatinine, eGFR, and albuminuria) could be found. On the other hand, analysis of influencing factors after transplantation (donor age, sum of HLA mismatches, and length of cold ischemia time) showed a deteriorated function both through donor age and duration of CIT.

#### BAFF-ELISA analysis

**Fig. 1:**
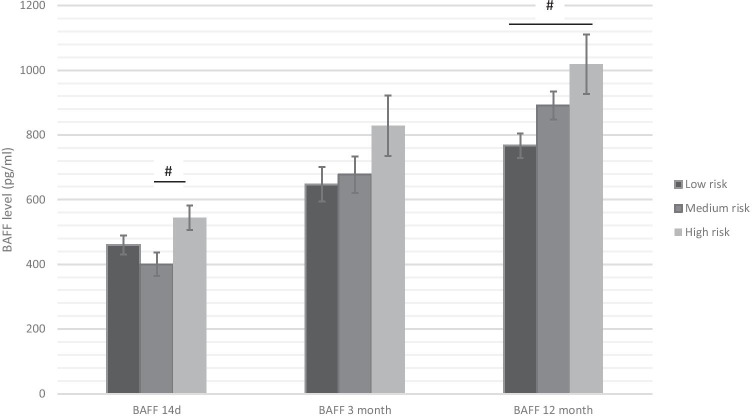
The BAFF level of the three groups over the observation period of 1 year. # =  < 0.05

BAFF levels at 14 d, 3 months, and 12 months after transplantation showed for patients with a low immunological risk profile initially a BAFF level of 459.9 pg/ml ± 189.7 pg/ml, which rose to 647.5 pg/ml ± 353.1 pg/ml (3 months) and then to 767.9 pg/ml ± 248.4 pg/ml (12 months). In the medium-risk group, the initial value was 400.1 pg/ml ± 212.1 pg/ml with an increase to 677.4 pg/ml ± 329.0 pg/ml (3 months) and further to 890.7 pg/ml ± 252.5 pg/ml (12 months). The BAFF starting level was 544.8 pg/ml ± 251.9 pg/ml in the group with the high immunological risk profile and then changed to 828.5 pg/ml ± 623.0 pg/ml (3 months) and 1018.8 pg/ml ± 610.5 pg/ml (12 months) (Fig. 1). Initially the BAFF expression level of the medium-risk patients were similar to the low-risk patients (p = 0.01 vs. high risk). In the follow-up, medium-risk patients displayed the most intense increase in BAFF expression level (123%) — which was even higher than in high-risk patients (87%) and low risk (67%). Thus, after 12 months medium-risk patients were almost comparable to high-risk patients, which displayed a significant difference to low risk (p = 0.04).

If one looked at the individual groups separately, it is noticeable that all patients from the medium risk group, who initially had BAFF values below the median, subsequently increased with their BAFF values after 3 months. No patient from this cohort showed BAFF values below the median at month 12. The situation is similar in the group with a high immunological risk. Only one patient was noticed with BAFF values below the median after 12 months.

#### Histological findings

All patients — even with a completely auspicious follow-up — received a protocol biopsy 14 d and 3 months after transplantation. Furthermore, indication biopsies were performed (e.g., for rise in creatinine, delayed graft function, new albuminuria, etc.). All biopsies were evaluated according to the BANFF 2017 criteria [16]. The results of the biopsies among the 3 groups and at different time points (0–30 d, 31–100 d, 101–365 d, > 365 d) were compared regarding incidence of any rejection and chronic lesions. When rejections occurred, they were either classified as an acute T cell-mediated rejection or as an antibody-mediated rejection according to the BANFF criteria, which were then also addressed therapeutically. Subclinical rejections in the sense of borderline rejections did not lead to any change in therapy if kidney function was stable. Borderline rejections were found in all three groups (low risk, n = 1; medium risk, n = 2; high risk, n = 1).

A look at the biopsies with regard to the lesions scores showed that the medium-risk group had a statistically more significant occurrence of chronic lesions in the sense of interstitial fibrosis (ci) and interstitial atrophy (ct) compared to the low-risk group (ci: biopsies < 30 days: p = 1.1 × 10^−8^; days 31–100: p = 1.2 × 10^−15^; biopsies up to day 365: p = 1.1 × 10^−09^; ct: biopsies < 30 days: p = 8.4 × 10 ^−8^; 31–100 days: p = 7.1 × 10^−19^; up to day 365: p = 1.1 × 10^−9^). Also in comparison to the high-risk group, the medium-risk group showed a trend towards an increased incidence of interstitial fibrosis and interstitial atrophy, reaching the level of significance in the biopsies, which were carried out between 31 and 100 days after the transplantation (ci: p = 0.02; ct: p = 0.02). If, on the other hand, one considers the occurrence of rejection at the mentioned time points, the group with medium risk showed no occurrence of T cell-mediated (TCMR) and only one antibody-mediated rejection (AMR).

Compared to this, a total of 8 rejections were detected in the low-risk group (2 AMR, 6 TCMR). A total of 9 rejections were detected in the group of patients with a high immunological risk (2 AMR, 7 TCMR).

#### De novo DSA development

According to the initial stratification, only patients in the high-risk group had performed donor-specific antibodies. In the follow-up after transplantation, patients in the low- and medium-risk groups only developed HLA class II de novo DSA. It was found that 4.5% of the patients (2 von 44) in the low-risk group and 8.8% of the medium group (3 of 34) developed de novo DSA, in contrast, to the high-risk group with 22.7% dnDSA positive patients (10 of 44).

### Stratification according to BAFF median

In a second step, the BAFF median at the time point 14 days of all patients (n = 122) was determined (399.4 pg/ml), and patients were stratified according to BAFF expression level: below vs. above BAFF median. Analysis of the baseline data showed that the donors of the cohort below the BAFF median were comparatively older (57 vs. 50 years, p = 0.006), whereas warm ischemia time was longer in patients above the BAFF median (14 min vs. 24 min, p = 0.03). All the other parameters showed no statistical difference.

The cohort above the BAFF median was more frequently induced with thymoglobulin (1.4 vs. 1.9, p = 0.007). Only one statistically significant difference could be observed in maintenance therapy, in the follow-up patients with BAFF expression levels above the median received higher steroid doses after one year. (2.8 vs. 4.4 mg, p = 0.04).

As in the first stratification, there was no relevant difference in resulting graft function (creatinine, eGFR, albuminuria) between the two groups during the observation period.

**Fig. 2:**
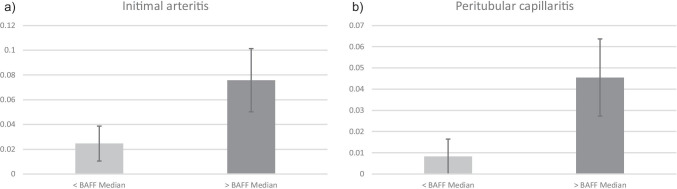
**a** Difference according to BAFF median at 14 days related to the occurrence of intimal arteritis. **b** Difference according to BAFF median at 14 days related to the occurrence of peritubular capillaritis

The histological analysis with regard to chronic lesions also showed no difference with the exception of focal sclerosis (p = 0.03) which was more intensified in the group above the median. In contrast, it was seen that patients with BAFF values above the median showed a marked trend towards more acute microvascular inflammation, in particular peritubular capillaritis (p = 0.07) and intimal arteritis (p = 0.09), all of which may be regarded as histopathological signs of AMR (Fig. 2 a and b). In contrast, lesions being associated with TCMR, e.g., interstitial inflammation and tubulitis differed not between both groups. When analyzing the biopsies for the occurrence of rejection, in accordance to the abovementioned observation, it was found that patients above the BAFF median suffered more often from rejections, especially antibody-mediated rejections (total: 6 vs. 12, ABMR: 1 vs. 4) (Table 3).

**Table 3:** Incidence and type of rejection episodes according to BAFF median at 14 day

	Below BAFF median				Above BAFF median			
	0–30 days postTx	30–100 days postTx	100–365 days postTx	> 365 days postTx	0–30 days postTx	30–100 days postTx	100–365 days postTx	> 365 days postTx
Rejection- total (n)	6				12			
TCMR (n)	0	1	0	4	4	2	2	0
ABMR (n)	1	0	0	0	3	0	1	0

Abbreviations: *TCMR*, T cell-mediated rejection, *ABMR* antibody-mediated rejection

In significantly more patients in the group above the median BAFF, preTX DSA (18 (30.5%) vs. 6 (9.5%)) could be detected. Seven patients (11.1%) in the group below the median developed de novo DSA, whereas in the group above the median, 8 patients (13.6%) were noticed with de novo DSA. Differences in the strength of the cumulative MFI values were not found in a significant range. However, at the time point of 12 months, there was a trend towards higher cumulative MFI values in the group above the median (p = 0.09).

Additionally, patients with a combination of BAFF values above the median and simultaneous approval of any DSA (either preTx or dnDSA) showed a slight increase in any rejection episodes (either TCMR or AMR) compared to patients with low BAFF values and DSA (21.4 vs. 14.3%). Similar findings were made by comparing patients with high BAFF values and the presence of DSA compared to no DSA evidence (21.4 vs. 6.7%).

## Discussion

In our current study, BAFF correlates well with the immunological risk profile before transplantation. Patients with a low and medium immunological risk profile started with low BAFF values, whereas patients with a high immunological risk stand out from the start due to increased BAFF values. In the further course of time, however, especially patients with medium risk turned out with the most marked increase in BAFF expression level. At 12 month, this group almost approaches the BAFF values of the high group — keeping in mind that, according to our stratification, both groups receive the same doses of maintenance immunosuppression.

A study showed that pre-transplant BAFF levels represent the extent of pre-immunization of transplant candidates. In contrast, a connection between the post-transplant BAFF levels and the occurrence of rejections, donor-specific antibodies, or clinical outcome could no longer be found [17].

According to our initial stratification, only high-risk patients showed preTX donor-specific antibodies. In the follow-up, mainly patients with high risk developed de novo DSA. It was also striking that the other two groups rarely developed de novo DSA and, above all, no HLA class I antibodies were found. The development of donor-specific antibodies and the occurrence of antibody-mediated rejection was demonstrated in three patients. Looking at the stratification according to the BAFF median, more preTx DSA were found in the group with patients above the BAFF median. This group tended to develop more de novo DSA in the following time. Additionally, the combination “high BAFF + DSA” exposed patients for an increased risk of allograft rejection (21.4 vs. 14.3% “low BAFF + DSA”).

In literature, there are different observations regarding the relationship between BAFF and the development of DSA. In their work, Slavcec found no correlation between increased BAFF values and the increased occurrence of de novo donor-specific antibodies [18]. In contrast, in the work of Thibault et al., de novo DSA can be detected in transplant patients with high BAFF levels and unstable kidney function [9]. This observation can be partially understood in our work. Patients with stable function and high BAFF values were more likely to develop de novo DSA than patients with low BAFF levels.

When analyzing the rejection profile, patients with medium immunological risk showed the fewest rejection episodes. Two antibody-mediated rejections were detected in the high-risk group. However, according to the BAFF levels after 14 days, it was seen that high BAFF levels were associated with increased rejection rates, in particular antibody-mediated rejection. In line with this observation, this patient group also showed a trend towards more microvascular inflammation in the sense of peritubular capillaritis and intimal arteritis in the further analysis of the histological lesions score. Patients with rejections also had significantly higher BAFF values at 14 days and 3 months than patients without the presence of a rejection.

This finding is in agreement with some of the works that can be found in literature. Sango et al. were able to show that the detection of histological changes, which represent antibody-mediated rejection, is associated with increased BAFF levels [19]. In their work, Wang and colleagues were also able to find increased BAFF levels in patients with acute rejection compared to a collective with stable function after transplantation [20]. They observed both cohorts over a period of 6 months. In the work of Irure-Ventura et al., increased BAFF values before transplantation were associated with an increased occurrence of antibody-mediated rejection in the first 12 months [21]. In contrast to these examinations and the results of our cohort, the patients with ABMR in the work of Slavcec stood out due to the low BAFF level. The authors suspect that this is due to the increased binding of BAFF to its receptors [18]. In contrast, Snanoudj et al. found no connection between BAFF and the occurrence of a rejection [22].

Xu et al. found increased BAFF values in their work, especially in patients with impaired kidney function compared to patients with stable function [23]. We could not observe this in our collective, since all patients showed stable function regardless of the BAFF levels over time. However, it is important to mention that BAFF — only with the exception of the work by Xu et al. [23] — has not been used as a parameter to assess graft function so far. It is rather interesting in the context of characterizing patients changing immunological risk after transplantation and thereby ensuring that the usage of an appropriate maintenance immunosuppression prevents rejection episodes. Patients with a medium risk showed the greatest increase in their BAFF values over time (123%) and nevertheless showed stable kidney function and few rejection episodes. A reduction in immunosuppression to the level of low-risk patients would therefore possibly be associated with an even greater increase in the BAFF values. This could result in a significantly higher risk of clinically relevant rejection episodes and a deterioration in graft function.

In our study, a significant change in BAFF levels in the group with medium immunological risk could be demonstrated. After 12 months, a risk profile similar to that of high-risk patients can be assumed. By adding the observation that patients with high BAFF values are at risk for an increased occurrence of microvascular inflammation and even severe rejections, BAFF can be used as an awareness factor. In this special patient population, the initial immunological risk allows a non T cell depleting induction therapy with Basiliximab as used in the low-risk group in comparison to thymoglobulin. Over time, however, an intensified maintenance immunosuppression, similar to those patients with a high risk, is indicated in order to balance the ongoing or aggravated immunological risk as reflected by changed BAFF values over time.

## Conclusion

In this cohort of highly standardized kidney transplant recipients, BAFF reflects the clinically defined underlying immunological risk profile after kidney transplantation very well. Furthermore, higher BAFF values are associated with an intensified risk for rejection, especially antibody-mediated rejection. Patients with a medium immunological risk profile show a significant increase in BAFF values in the course after transplantation, so that a higher immunosuppressive therapy compared to low-risk patients is justified to relativize this changed immunological risk and BAFF can be used as an awareness factor for these patients over time.

## Supplementary Information

Below is the link to the electronic supplementary material.

ESM 1 (DOCX 17 kb)

ESM 2 (DOCX 18 kb)
