# Supplementary material for: B-cell activating factor BAFF as a novel alert marker for the immunological risk stratification after kidney transplantation
Source: Immunol Res. 2021 Aug 10;69(6):487–95. doi: 10.1007/s12026-021-09205-4 (PMC8580904; doi:10.1007/s12026-021-09205-4)
Supplement: Supplementary file 1 — (DOCX 17 kb) [file 12026_2021_9205_MOESM1_ESM.docx]

|  | **Low risk** | **Medium risk** | **High risk** |
| --- | --- | --- | --- |
| **Risk Stratification criteria*** | - First transplant - CDC-PRA < 5% - AB0-compatible KTx - No DSA | - CDC-PRA 5-30% - HLA-antibodies, but no DSA - Re-KTX without an early immunological graft loss in previous KTx | - CDC-PRA > 30% - Re-KTx with an early immunological graft loss in previous KTx - Re-KTX with repeat HLA –Mismatch |
| **Induction therapy** | anti-CD25-antibody (Basiliximab) | anti-CD25-antibody (Basiliximab) | T-cell-depleting antibody (Thymoglobulin) |
| **Maintenance therapy** | 1. Tacrolimus 2. Mycophenolat acid 3. Steroids-> Tapering off after 3 months except for GN as the underlying disease | 1. Tacrolimus 2. Mycophenolat acid 3. Steroids-> Tapering off after 3 months except for GN as the underlying disease | 1. Tacrolimus 2. Mycophenolat acid 3. Steroids 5mg (maintenance) |

Suppl. Table 1. Immunological criteria for risk stratification before transplantation. ***If one criterion is met, this is sufficient for placement in risk group**

|  | **Low risk** | **Medium risk** | **High risk** |
| --- | --- | --- | --- |
| **Tacrolimus** | No difference | | |
| **Mycophenolat acid** | Initial dose: 2 x 720 mg/d  After week 4: 2 x 540 mg/d  After 12 month: 2 x 360mg/d | No reduction in dosage (2 x 720mg) | |
| **Cellcept** | Initial dose: 2 x 1000 mg/d  After week 4: 2 x 750 mg/d  After 12 month: 2 x 500mg/d | No reduction in dosage (2 x 1000mg) | |
| **Steroids** | Tapering off over 3 months except for GN as the underlying disease | No medication leakage (5 mg maintenance) | |

Suppl. Table 2. Differences in the immunosuppressive regime
