# Supplementary material for: B-cell activating factor BAFF as a novel alert marker for the immunological risk stratification after kidney transplantation
Source: Immunol Res. 2021 Aug 10;69(6):487–95. doi: 10.1007/s12026-021-09205-4 (PMC8580904; doi:10.1007/s12026-021-09205-4)
Supplement: Supplementary file 2 — (DOCX 18 kb) [file 12026_2021_9205_MOESM2_ESM.docx]

|  | **Low risk (n=44)** | | | | |
| --- | --- | --- | --- | --- | --- |
|  | 14d postTx (n=44) | 3 months postTx (n=43) | 12 months postTx (n=41) | 2 years postTx (n=32) | 3 years postTx (n=17) |
| creatinine (mg/dl) | 2.6 $\pm$ 1.8 | 1.7$\pm$ 0.7 | 1.7$\pm$ 0.8 | 1.9 $\pm$ 1.2 | 1.6 $\pm$ 0.8 |
| eGFR | 35.1$\pm$ 17.0 | 47.3$\pm$ 16.2 | 51.3$\pm$ 20.4 | 48.5$\pm$ 22.4 | 50.6$\pm$ 20.6 |
| albuminuria (mg/g crea) | 252.0$\pm$ 350.0 | 130.0$\pm$ 385.1 | 144.3$\pm$ 392.1 | 150.7$\pm$ 386.1 | 37.4$\pm$ 41.9 |

Suppl. Table 3a. Clinical outcome data of the low risk group.

|  | **Medium risk (n=34)** | | | | |
| --- | --- | --- | --- | --- | --- |
|  | 14d postTx  (n=34) | 3 months postTx (n=34) | 12 months postTx (n=34) | 2 years postTx (n=28) | 3 years postTx (n=17) |
| creatinine (mg/dl) | 2.2 $\pm$ 0.9 | 1.7$\pm$ 0.4 | 1.7$\pm$ 0.6 | 1.8 $\pm$ 1.0 | 1.7 $\pm$ 0.6 |
| eGFR | 35.8$\pm$ 14.6 | 43.6$\pm$ 12.8 | 48.5$\pm$ 16.9 | 50.4$\pm$ 19.7 | 49.1$\pm$ 20.2 |
| albuminuria (mg/g crea) | 186.5$\pm$ 256.6 | 75.6$\pm$ 177.8 | 112.8$\pm$ 303.7 | 498.6$\pm$ 1979.5 | 118.6$\pm$ 181.5 |

Suppl. Table 3b. Clinical outcome data of the medium risk group.

|  | **High risk (n=44)** | | | | |
| --- | --- | --- | --- | --- | --- |
|  | 14d postTx (n=44) | 3 months postTx (n=44) | 12 months postTx (n=43) | 2 years postTx (n=32) | 3 years postTx (n=13) |
| creatinine (mg/dl) | 2.5 $\pm$ 1.7 | 1.6$\pm$ 0.6 | 1.6$\pm$ 0.7 | 1.7$\pm$ 1.1 | 2.1 $\pm$ 1.7 |
| eGFR | 35.9$\pm$ 20.5 | 49.2$\pm$ 19.0 | 51.3$\pm$ 21.2 | 53.4$\pm$ 23.8 | 47.2$\pm$ 26.9 |
| albuminuria (mg/g crea) | 185.0$\pm$ 338.0 | 73.8$\pm$ 116.7 | 44.5$\pm$ 59.8 | 52.7$\pm$ 55.8 | 52.9$\pm$ 76.3 |

Suppl. Table 3c. Clinical outcome data of the high risk group.
